# Transcriptomic analysis of the differentiating ovary of the protogynous ricefield eel *Monopterus albus*

**DOI:** 10.1186/s12864-017-3953-6

**Published:** 2017-08-03

**Authors:** Jinfeng Cai, Wei Yang, Dong Chen, Yize Zhang, Zhi He, Weimin Zhang, Lihong Zhang

**Affiliations:** 1 Department of Biology, School of Life Sciences, Sun Yat-sen University, Guangzhou, 510275 People’s Republic of China; 2 Institute of Aquatic Economic Animals and Guangdong Province Key Laboratory for Aquatic Economic Animals, Sun Yat-Sen University, Guangzhou, 510275 P. R. China; 3 College of Animal Sciences and Technology, Sichuan Agricultural University, Ya’an, 625014 P. R. China

**Keywords:** *Monopterus albus*, Ovary, Transcriptome, Differentiation, Meiosis

## Abstract

**Background:**

The ricefield eel is a protogynous hermaphroditic Synbranchiform species that changes sex naturally from female to male, which offers an interesting model for studying gonadal (particularly ovarian) differentiation in vertebrates. In the present study, transcriptome sequencing of the gonad of ricefield eel larvae was performed to explore the molecular mechanisms underlying the ovarian differentiation and development.

**Results:**

A total of 301,267,988 clean reads were generated from cDNA libraries of gonadal tissues of ricefield eel larvae at 6, 9, 12, and 20 days post hatching (dph), which contained undifferentiated gonads, differentiating ovaries, ovaries with oogonia, and ovaries with meiotic oocytes, respectively. De-novo assembly of all the clean reads generated a total of 265,896 unigenes with a mean size of 720 bp and a N50 of 1107 bp. RT-qPCR analysis of the developmental expression of 13 gonadal development-related functional genes indicated that RNA-seq data are reliable. Transcriptome data suggest that high expression of female development-related genes and low expression of male development-related genes in the early gonads of ricefield eel larvae participate in the cascade of sex differentiation leading to the final female phenotype. The contrasting expression patterns of genes involved in retinoid acid (RA) synthesis and degradation might result in peak production of RA at 12 dph in the gonad of ricefield eel larvae, and induce molecular events responsible for the initiation of meiosis before the meiotic signs could be observed at 20 dph. In addition, only *stra6* but not *stra8* could be identified in gonadal transcriptome data of ricefield eel larvae, and the expression pattern of *stra6* paralleled those of genes involved in RA synthesis, suggesting that *stra6* may be a downstream target of RA and play a role in RA metabolism and/or meiotic initiation in the gonad of ricefield eel larvae.

**Conclusions:**

The present study depicted the first large-scale RNA sequencing of the gonad of ricefield eel larvae, and identified many important functional genes, GO terms and KEGG pathways involved in gonadal development and germ cell meiosis. Results of the present study will facilitate future study on the ovarian differentiation of ricefield eels and other teleosts as well.

**Electronic supplementary material:**

The online version of this article (doi:10.1186/s12864-017-3953-6) contains supplementary material, which is available to authorized users.

## Background

Gonadal development of vertebrates relies on two basic interacting processes: sex determination (SD) and gonad differentiation (GD). SD leads to a binary fate choice of the sexual characteristics in an organism. On the other hand, GD leads to the formation of an ovary or a testis once the fate of the undifferentiated gonad has been determined [1]. In mammals, testis is determined by *Sry* gene on Y chromosome, which acts in fetal gonads and induces development of testes rather than ovaries. Besides, *Sox9* is directly activated by *Sry* and is responsible and sufficient for fating the male gonad in mammals [2]. However, the primary sex-determining decision is not final in females or males. Loss of FOXL2 in adult granulosa cells reprograms granulosa cells into Sertoli cells [3]. On the other hand, *Dmrt1* is essential to maintain mammalian testis determination, and competing regulatory networks are involved in the maintenance of gonadal sex long after the fetal choice between male and female [4]. In teleosts, many genes and their interactions have been reported to be involved in SD and GD recently [5]. *Dmy* is shown to be a prime candidate for the sex-determining gene in *Oryzias latipes* and in *Oryzias curvinotus* [6]. *GsdfY* (gonadal soma derived growth factor on the Y chromosome) has replaced *Dmy* as the master sex-determining gene in *Oryzias luzonensis* [7]. Other master sex-determining genes, including *amhy* in *Odontesthes hatcheri* [8], *amhr2* in *Takifugu rubripes*, *T. pardalis* and *T. poecilonotus* [9], *sdY* in *Oncorhynchus mykiss* [10], have also been reported. Therefore, fish exhibit a high diversity of sex determining mechanisms, which in most species remain largely unknown. Downstream sex determination, genes involved in gonadal differentiation in teleosts are fairly conserved, including *dmrt1* and *amh* in testis differentiation and *cyp19a1a* and *foxl2* in ovarian differentiation [11].

During early ovarian development in mammals, the accumulation of retinoic acid (RA) stimulates Stra8 to activate factors that promote meiosis of oogonia [12]. The intracellular level of active RA is determined by the balance between its synthesis by RALDHs and its degradation by CYP26 enzymes [13]. In chickens and salamanders, Aldh1a2 and Cyp26b1 are involved in meiotic initiation of the germ cells [14, 15]. In teleosts such as zebrafish [16] and tilapia [17], Aldh1a2 and Cyp26a1 regulate the homeostasis of RA and play critical roles in meiotic initiation. These studies suggest that the metabolism and roles of RA may be conserved in meiosis of teleosts although Stra8 has not been identified in most teleost species except Southern catfish [18].

Ricefield eel (*Monopterus albus*), a member of the order Synbranchiformes, is a protogynous hermaphrodite fish that undergoes sex change from female to male through an intersexual phase during its life cycle. This sex changing phenomenon has attracted a lot of research interests, and many sexual differentiation-related genes have been analyzed in ricefield eels, including *cyp19a1a* [19], *gdf9* [20], *lhb* and *fshb* [21], *amh* and *dax1* [22]. The possible involvement of epigenetic mechanisms, such as DNA methylation of *cyp19a1a* gene [23] and gonadal miRNAs [24], in the sex change of ricefield eels was also examined. Besides sex change, the uni-directional gonadal development towards the ovary in ricefield eels is also intriguing and offers a very good model for analyzing the molecular mechanisms underlying the ovarian differentiation in teleosts. Previously, we have analyzed the gonadal development of ricefield eel larvae through histological observation, and shown that the indifferent gonad differentiated into the ovary at 7 dph [25]. However, the regulatory gene networks and mechanisms of ovarian differentiation in ricefield eels remain to be explored.

The next-generation high-throughput sequencing technology has been employed to identify genes involved in gonadal development, sex determination and sex differentiation [26, 27]. In the present study, large-scale transcriptomic analysis of gonads of ricefield eel larvae around ovarian differentiation was performed with the intention of revealing the genes and gene networks involved in the early ovarian differentiation. It is suggested that up-regulation of female-related genes and low expression of male-related genes in gonads of ricefield eel larvae participate in the cascade of sex differentiation leading to the final female phenotype. The up-regulation of genes for synthesis of retinoid acid (RA) and low expression of genes for RA degradation may be a pre-requisite for adequate production of RA and subsequent initiation of meiosis in the differentiated ovaries of ricefield eel larvae. Results of present study provide a solid foundation for further elucidation of the mechanisms of the ovarian differentiation in ricefield eels and other teleosts as well.

## Methods

### Experimental fish and sample collection

The adult ricefield eels (females:body length 35–45 cm and body weight 35–60 g;males: body length 45–60 cm and body weight 90–250 g) were purchased from Dazhong Breeding Co. Ltd. (Jianyang, Sichuan, China), and raised in outdoor concrete ponds under natural photoperiod (30·39°N) and temperature (21.5–32.5 °C) feeding on live *Chironomus* sp. larvae. In May 2015, mature male and female fish were selected for mating and newly hatched ricefield eel larvae were obtained for the present study. The ricefield eel larvae were fed with finely tubificidae starting at 5 days post hatching (dph), and were collected at 6, 9, 12, and 20 dph for analysis, with the average body length (cm) of 2.44 ± 0.15, 3.31 ± 0.09, 4.07 ± 0.14 and 5.17 ± 0.14, respectively, and average body weight (g) of 0.0126 ± 0.0017, 0.0197 ± 0.0018, 0.0392 ± 0.0032 and 0.0600 ± 0.0047, respectively. At each time point, 3 larvae were fixed in Bouin solution for 24 h and then stored in 70% ethanol until histological examination of gonadal stages, and 6 larvae were kept in Sample Protector for RNA/DNA (Takara Biotechnology, Dalian, China) for the extraction of tissue RNA.

### Histological examination of gonadal stages of ricefield eel larvae

The Bouin-fixed ricefield eel larvae were decalcified in Fisher’s Cal-Ex solution (Thermo Fisher Scientific, USA) overnight and then washed with running tap water for 24 h before conventional histological processing. The whole body of ricefield eel larvae was embedded in paraffin. Serial cross sections were cut at 5 μm and stained with haematoxylin/eosin. Micrographs were taken under a microscope (E800, Nikon, Japan).

### RNA extraction from gonadal tissues of ricefield eel larvae

The ricefield eel larvae were kept in Sample Protector for RNA/DNA (Takara) and the gonadal tissue was dissected under a dissecting microscope (SteREO Lumar, V12, Zeiss, Germany. Additional file 1: Figure S1). Gonadal tissues were pooled together from 4 individuals at 6 and 9 dph, or from 3 individuals at 12 and 20 dph, and three to five pool replicates were obtained for each stage. The total RNA was isolated from the gonadal tissues using the E.Z.N.A. microElute Total RNA Kit (Omega Bio-tek, USA) by following the manufacturer’s instructions. Then RNA quality was determined with a 2100 Bioanalyser (Agilent, CA, USA) and quantified using a ND-2000 (NanoDrop Technologies, DE, USA). To ensure that the RNA obtained is indeed from gonadal tissues, RT-PCR analysis was performed to examine the expression of *vasa* (DQ174775) and *bactin* (AY647143) using gene-specific primers (Additional file 2: Table S1). The former was shown to be exclusively expressed in gonads of ricefield eels [28]. Briefly, 100 ng RNA was treated with RNase-free DNase I (Omega Bio-tek, USA) to remove any genomic DNA contamination and then reverse transcribed (RT) with the RevertAid H Minus First Strand cDNA Synthesis Kit (Fermentas, UAB, USA) by using random primers according to the manufacturer’s instructions.

The first-strand cDNA synthesized above (0.25 μl) was amplified for *vasa* and *bactin* genes using a TGRADIENT thermocycler (Biometra GmbH, Germany). Polymerase chain reaction (PCR) was performed in a 25 μl final volume, which contained 2.5 μl of 10× *Taq* buffer, 2.0 mM MgCl2, 0.2 mM deoxynucleotide triphosphate (dNTP), 0.4 μM of each primer and 1.25 U Taq DNA Polymerase (Fermentas UAB, USA). Water was used as a negative control in RT-PCR analysis. The reaction mixture was heated at 94 °C for 3 min, followed by amplification of 34 cycles for target genes. The cycling conditions were 94 °C for 0.5 min, 56 °C for 0.5 min and 72 °C for 1 min, with a final extension at 72 °C for 10 min. The PCR products were separated on a 1.5% agarose gel and visualized by ethidium bromide staining. The gel image was captured on the Bio-Rad GelDoc 2000 (BioRad Laboratorie, CA, USA).

### Library construction and Illumina Hiseq2500 sequencing

A total amount of 1.5 μg RNA per sample was used as input material for the RNA sample preparation. Sequencing libraries were constructed using the NEBNext® Ultra™ RNA Library Prep Kit for Illumina® (NEB, USA) according to manufacturer’s instructions, and index codes were added to attribute sequences to each sample. Briefly, mRNA was purified from total RNA using poly-T oligo-attached magnetic beads, fragmented using divalent cations under elevated temperature in NEBNext First Strand Synthesis Reaction Buffer (5X), and followed by first strand cDNA synthesis using random hexamer primer and M-MuLV Reverse Transcriptase (RNase H-). Second strand cDNA synthesis was subsequently performed using DNA Polymerase I and RNase H, and followed by blunting remaining overhangs via exonuclease/polymerase activities. After adenylation of 3′ ends of DNA fragments, NEBNext adaptors with hairpin loop structures were ligated to prepare for hybridization. cDNA fragments of preferentially 150 ~ 200 bp in length were selected by purifying the library fragments with the AMPure XP system (Beckman Coulter, Beverly, USA). Then 3 μl USER Enzyme (NEB, USA) was used with size-selected, adaptor-ligated cDNA at 37 °C for 15 min followed by 5 min at 95 °C before PCR. Then PCR was performed with Phusion High-Fidelity DNA polymerase, Universal PCR primers and Index (X) Primer. Finally, PCR products were purified with the AMPure XP system and library quality was assessed with the Agilent Bioanalyzer 2100 system.

The clustering of the index-coded samples was performed on a cBot Cluster Generation System using TruSeq PE Cluster Kit v3-cBot-HS (Illumia) by following the manufacturer’s instructions. After cluster generation, the libraries were sequenced on an Illumina Hiseq platform and paired-end reads were generated.

### De-novo assembly and functional annotation

Raw data (raw reads) of fastq format were firstly processed through in-house perl scripts, where clean data (clean reads) were obtained by removing reads containing adapters, reads containing ploy-N and low quality reads from raw data. At the same time, Q20, Q30, GC-content and sequence duplication levels of the clean data were calculated. All the downstream analysis was based on clean data of high quality. The Trinity method (version r20140413p1) [29] was used for the de novo assembly of the clean data from samples with the minimum kmer_cov set to 2 as the default, and all other parameters set to default. To avoid redundant transcripts, TGICL (version 2.1) [30] were applied to extract the longest transcripts as unigenes. Unigenes generated with the assembly were used for downstream analysis. After sequence assembly, unigenes were used for BLASTN searches with annotation against the Nt database using an E-value cut-off of 10^−5^ (E-value <0.00001), and also aligned by BLASTX to protein databases such as SwissProt, KEGG and COG, in order to retrieve proteins with the highest sequence similarity to the given unigenes along with putative functional annotations. Gene function was annotated based on the following databases: Nr, Nt, Pfam, KOG/COG, SwissProt, KO and GO.BLAST2GO program (version b2g4pipe_v2.5) was used to retrieve GO annotations of unigenes for describing biological processes, molecular functions and cellular components. Metabolic pathway analysis was performed using the online KEGG Automatic Annotation Server.

### Gene expression analysis

The expected number of Fragments Per Kilobase of transcript sequence per Millions base pairs sequenced (FPKM) values were calculated for the gene expression level. FPKM >0.3 was used to identify whether a gene is expressed or not.

Prior to differential gene expression analysis, for each sequenced library, the read counts were adjusted by edger program package (version 3.0.8) through one scaling normalized factor. A differential gene expression analysis of pairs of samples (9 dph vs. 6 dph, 12 dph vs. 9 dph, and 20 dph vs. 12 dph) was performed using the DEGseq R package (version 1.12.0) [31]. *P* value was adjusted using q value. The q value is a measure of significance in terms of the false discovery rate rather than the false positive rate. A systematic use of q values in genomewide tests of significance will yield a clear balance of false positives to true positive results and give a standard measure of significance that can be universally interpreted [32]. Q value < 0.005 & |log2(fold change)| > 1 was set as the threshold for significantly differential expression.

### Validation of differentially expressed genes from transcriptome with real-time PCR

The relative mRNA levels of 13 differentially expressed genes identified from transcriptome were examined by quantitative real-time PCR (qPCR) to verify their expression profiles in gonads of ricefield eel larvae at 6, 9, 12, and 20 dph, respectively. Seven of the 13 genes are related to female gonadal differentiation, three to male gonadal differentiation, two to RA synthesis, and one to RA degradation. The main purpose of analyzing the expression of these 13 genes by qPCR is to test if their expression profiles parallel with those inferred from RNAseq data. Three to five biological replicates were employed for each stage. Total RNA (100 ng) from each sample was reverse transcribed with random primers by using the RevertAid H Minus First Strand cDNA Synthesis Kit (Fermentas UAB, USA) according to the manufacturer’s instruction. The qPCR reaction was performed in a 10 μl reaction volume using the Roche LightCycler 480 with SYBR Green PCR master mix (DBI® Bioscience, Germany). The oligonucleotide primers (Additional file 2: Table S1) were designed to span exons when possible. The cycling parameters were: 95 °C for 5 min, followed by 40 cycles of amplification at 95 °C for 10 s, 58 °C for 15 s and 72 °C for 20 s. The specificity of qPCR amplification was confirmed by melt-curve analysis, agarose gel electrophoresis, and sequencing of PCR products.

The quantification of the mRNA expression level was conducted using a standard curve with tenfold serial dilutions of the plasmid containing the corresponding DNA fragment which ranges from 10^1^ to 10^8^ copies. The correlation coefficients and PCR efficiencies were not less than 0.950 and 80%, respectively. To minimize variation due to the differences in RNA loading, the geometric mean expression levels of *bactin*, *gapdh*, and *hprt1* were used to normalize the expression levels of the target genes.

## Results

### Isolation and confirmation of RNA samples from gonadal tissues of ricefield eel larvae

**Fig. 1:**
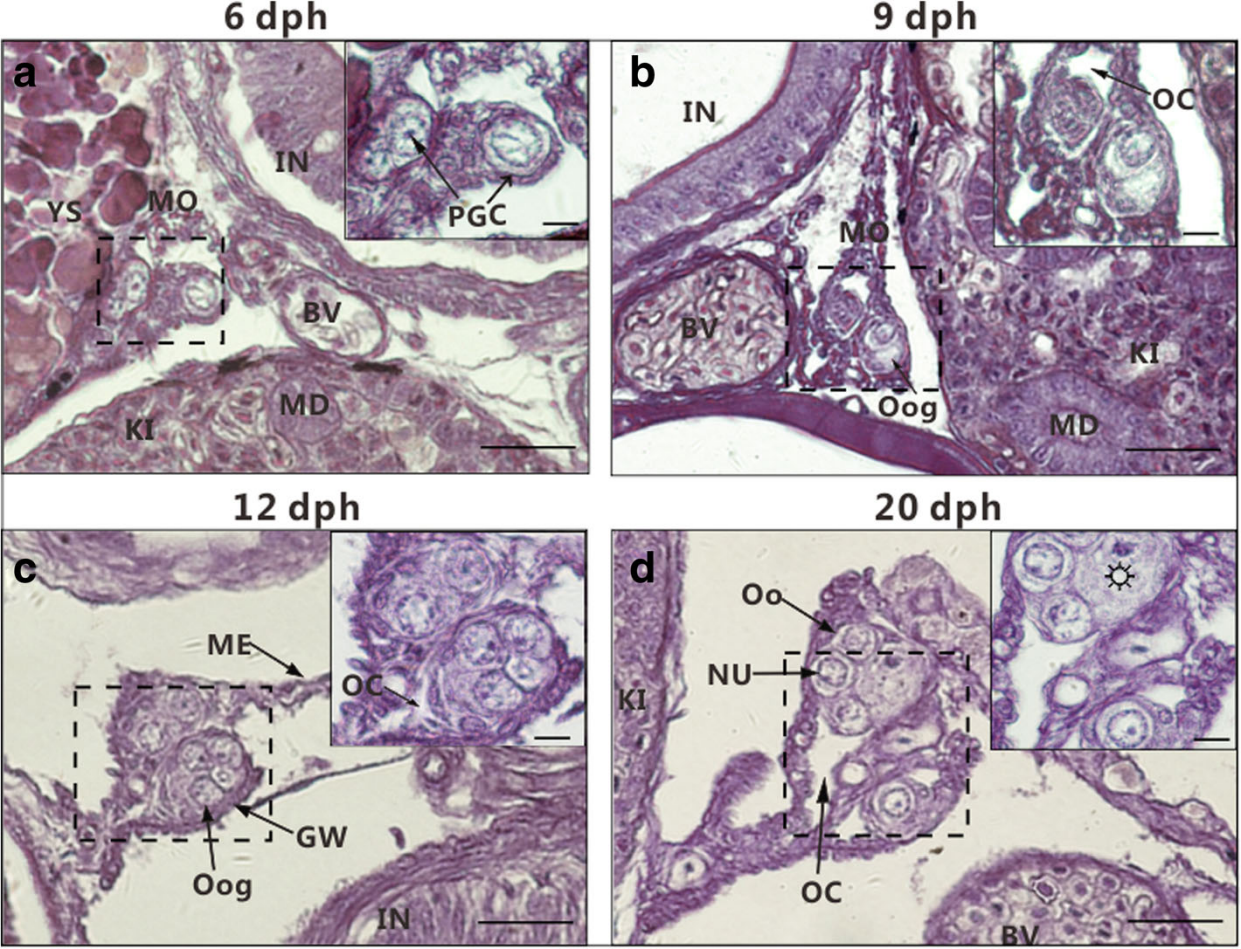
The gonadal histology of ricefield eel larvae. **a** The gonad at 6 dph. The primordial germ cell (PGC) was in isolated states. **b** The gonad at 9 dph. The ovarian cavities could be observed. **c** The gonad at 12 dph. The number of PGCs increased greatly and the majority of germ cells showed clear borders and most likely developed as oogonia. **d** The gonad at 20 dph. The cytoplasm of oogonium was inconspicuous, and the borders of nuclei were very clear. Some germ cells () containing condensate chromatins might have developed into pachytene oocytes. The inset is the magnification of the boxed area in each image (Scale bar = 5 μm). PGC, Primordial germ cell; BV, blood vessel; IN, intestine; YS, yolk sac; MD, mesonephric duct; KI, kidney; ME, mesentery; GW, gonadal wall; MO, mesogonium; NU, nucleolus; OC, ovarian cavity; Oog, oogonium; Oo, oocytes; dph, days post hatching; Scale bar = 25 μm except the insets

The gonadal histological images of ricefield eel larvae at 6, 9, 12, and 20 dph (Fig. 1) are similar to those observed in our previous report [25]. The gonads of ricefield eel larvae were undifferentiated at 6 dph (Fig. 1a), but differentiated as ovaries at 9 dph with the appearance of ovarian cavities (Fig. 1b). The germ cells developed as oogonia at 12 dph (Fig. 1c), then entered meiosis at 20 dph with the appearance of oocytes at the leptotene stage of prophase in meiosis I (Fig. 1d). The gonadal tissues (with an average diameter of 50 μm) of ricefield eel larvae were dissected out under a stereomicroscope (Additional file 1: Fig. S1), which was hardly discernible visually and presented a great challenge for the present study. RT-PCR analysis detected the expression of both *bactin* and *vasa* (Additional file 1: Fig. S2) in the isolated gonadal tissues, confirming the authenticity of gonadal tissues obtained.

### Transcriptome sequencing and de novo assembly

Four cDNA libraries were constructed with the respective total RNA extracted from gonadal tissues of ricefield eel larvae at 6, 9, 12, and 20 dph, and designated as HS6, HS9, HS12, and HS20, respectively. The cDNA libraries were sequenced on an Illumina Hiseq 2000 platform. A total of 378,352,620 raw reads were obtained, which produced a total of 301,267,988 clean reads after removing low-quality reads and adapter sequences (Table 1). All paired-end reads were pooled together and assembled de novo using Trinity and 312,062 transcripts with lengths ≥200 bp were obtained. The mean length of these transcripts was 909 bp, and the maximum length was 19,212 bp. The longest copy of redundant transcripts was regarded as a unigene, and the transcripts were assembled into 265,896 putative unigenes. Among all the putative unigenes, 18,373 unigenes had a length longer than 2000 bp, 23,909 unigenes between 1000 and 2000 bp, and 55,662 unigenes between 500 and 1000 bp (Additional file 1: Fig. S3A), which represented approximately 7.0%, 9.0%, and 21.0% of the total, respectively. The mean length of the putative unigenes was 720 bp, with a N50 of 1107 bp (Additional file 1: Fig. S3B).

**Table 1:** Summary of the sequence assembly after Illumina sequencing

Name	Raw reads	clean reads	Clean bases (Gb)	Error (%)	Q20 (%)	Q30 (%)	GC (%)
HS6_1	49,903,755	38,402,310	4.77	0.03	96.57	92.85	44.76
HS6_2	49,903,755	38,402,310	4.65	0.03	95.98	92.20	44.95
HS9_1	46,539,168	35,817,632	4.45	0.05	93.95	88.36	44.18
HS9_2	46,539,168	35,817,632	4.35	0.04	95.01	90.34	44.01
HS12_1	51,223,821	44,394,563	5.52	0.04	94.93	89.93	45.28
HS12_2	51,223,821	44,394,563	5.3	0.04	94.76	89.94	45.89
HS20_1	41,509,566	32,019,489	3.98	0.03	96.62	92.95	46.08
HS20_2	41,509,566	32,019,489	3.97	0.03	96.14	92.47	46.11
Total	378,352,620	301,267,988	36.97	-	-		

The numbers 1 and 2 at the end of the library name represent left and right ends (pair-end sequencing), respectively. Gb: Giga base; Q20: percentage of bases with a Phred value of at least 20; Q30: percentage of bases with a Phred value of at least 30

### Annotation of unigenes

The annotation of unigenes was based on their similarities with known or putative annotations in public databases, including GenBank Nr, GenBank Nt, SwissProt, KO, PFAM, GO, and KOG (E values ≤1e ^– 5^ for the former three databases; E values ≤1e ^− 3^ for the last database). Among the 265,896 high-quality unique sequences, 71,158 (26.76%) had at least one significant match with an existing gene model in BLAST searches (Table 2).

**Table 2:** Summary statistics of functional annotation for ricefield eel unigenes in public databases

Type	Number of Unigenes	Percentage (%)
Annotated in Nr	36,538	13.74
Annotated in Nt	48,487	18.23
Annotated in KO	15,239	5.73
Annotated in SwissProt	28,357	10.66
Annotated in PFAM	40,536	15.24
Annotated in GO	40,672	15.29
Annotated in KOG	17,756	6.67
Annotated in all Databases	8945	3.36
Annotated in at least one Database	71,158	26.76
Total Unigenes	265,896	100

*Nr* NCBI non-redundant protein sequences, *Nt* NCBI non-redundant nucleotide sequences, *KO* Kyoto encyclopedia of genes and genomes ortholog database, *SwissProt* A manually annotated and reviewed protein sequence database, *PFAM* Protein family, *GO* Gene Ontology, *KOG* Clusters of Orthologous Groups of proteins

**Fig. 2:**
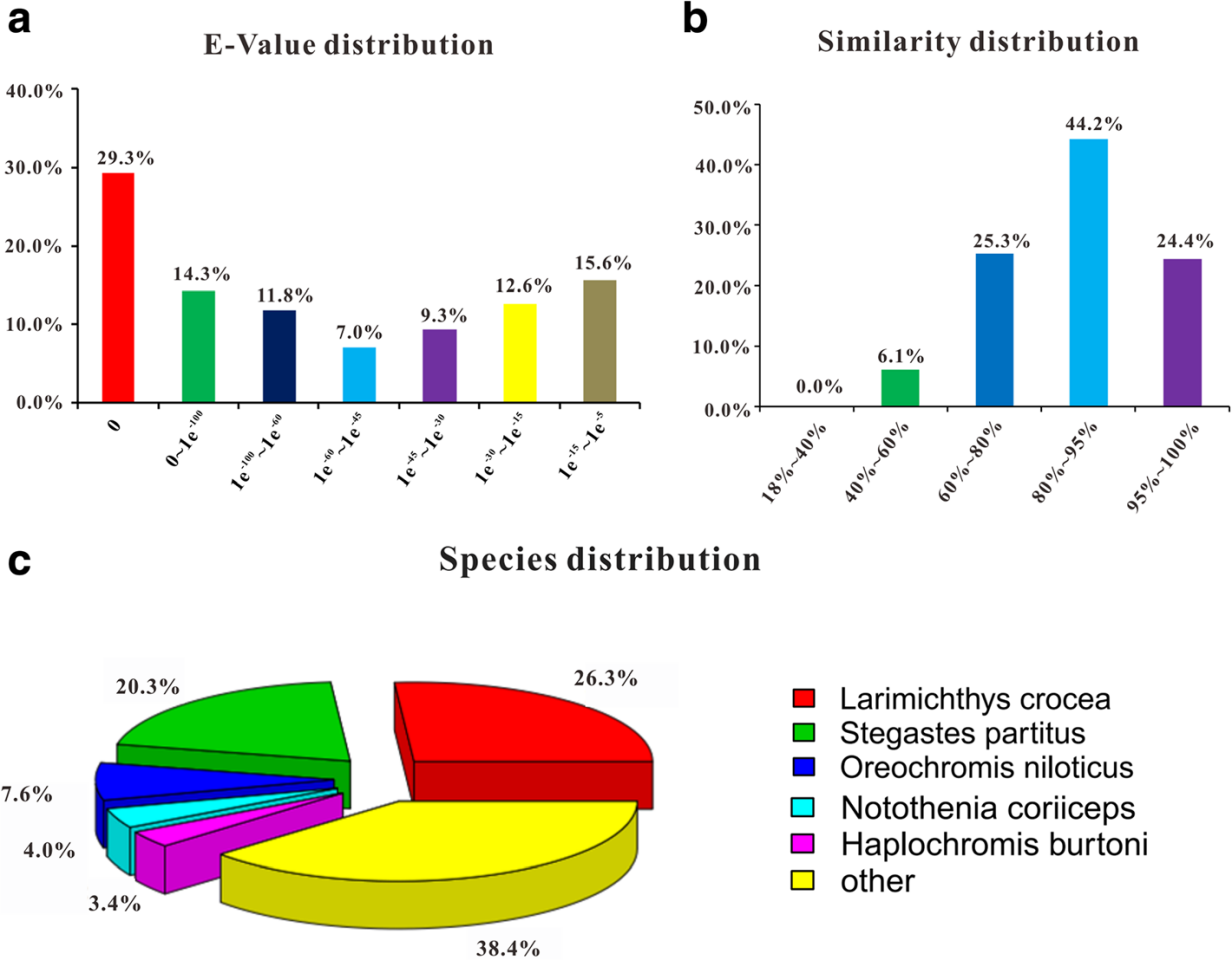
Homology analysis of the gonadal transcriptome of ricefield eel larvae. All distinct gene sequences that had BLAST annotations within the Nr database with a cutoff e-value ≤ 10^−5^ were analyzed for e-value distribution **a**, similarity distribution **b**, and species distribution **c**

The e-value distribution of the top hits in the Nr database showed that 43.6% of the mapped sequences have strong homology (<1e^−100^) whereas 56.4% of the homolog sequences ranged between 1e^−5^ and 1e^−100^ (Fig. 2a). For similarity distribution, 93.9% of the sequences showed a similarity >60% and the remaining 6.1% of the sequences had a similarity ranging from 40% to 60% (Fig. 2b). The species distribution showed that 26.3% of the matched unigenes shared highest similarities with *Larimichthys crocea*, followed by *Stegastes partitus* (20.3%), and *Oreochromis niloticus* (7.6%) (Fig. 2c).

**Fig. 3:**
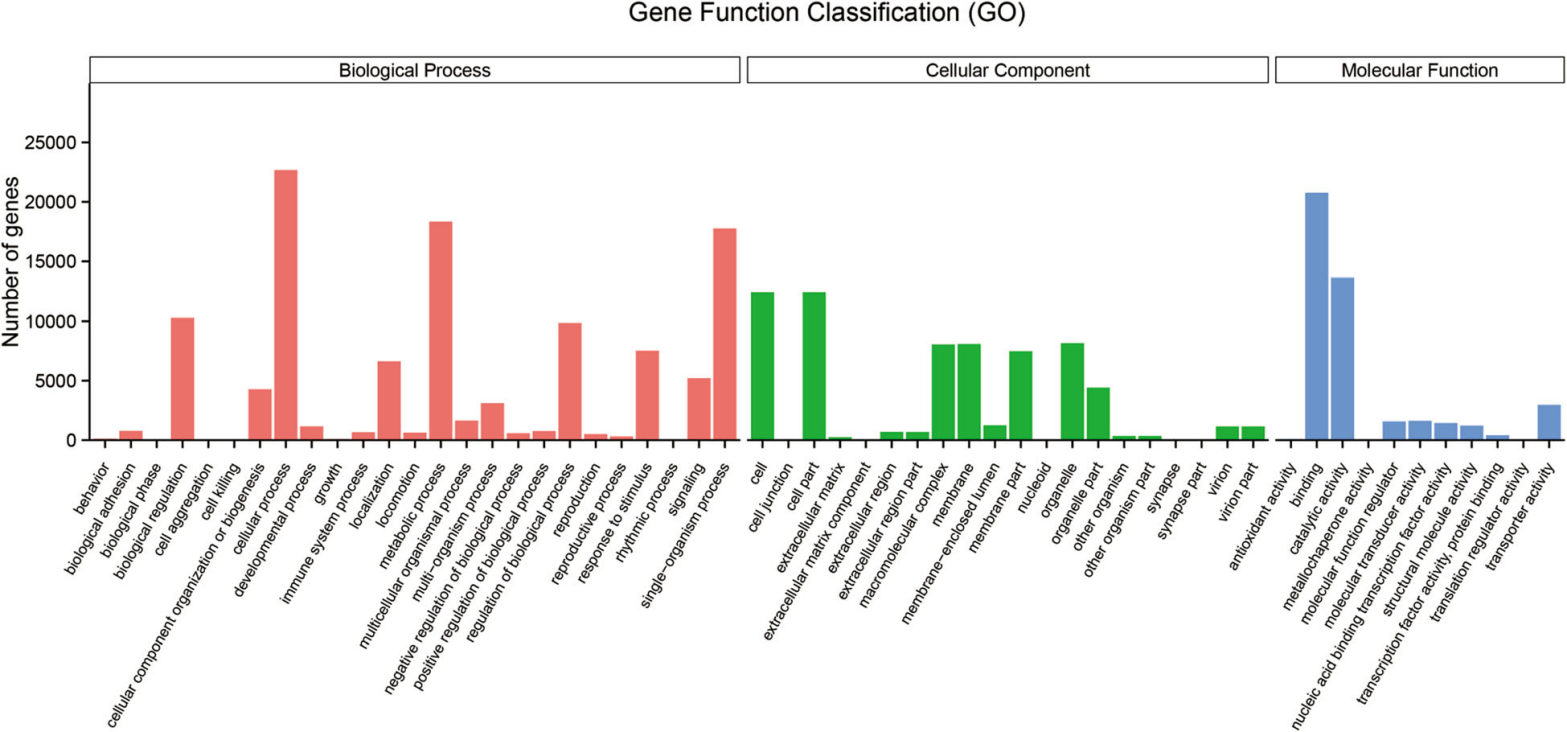
GO classification analysis of unigenes. GO functions are shown in *X* axis. The *Y* axis shows the numbers of genes which have the GO function

Gene ontology is a standardized system for gene functional classification, which includes biological process, cellular components and molecular functions of gene products. GO analysis of our dataset showed that a total of 40,672 (15.29%) unigenes were annotated (Table 2). The three main GO categories were classified into 56 subcategories (Fig. 3). At the biological process level, metabolic process (GO: 0008152), cellular process (GO: 0009987) and single-organism process (GO:0044767) rank among the first three categories. In the category of cellular components, most of the corresponding genes were involved in cell (GO: 0005623) and cell part (GO: 0044464). As for the molecular functions, the majorities of the GO terms were grouped into either binding (GO: 0005488) or catalytic activity (GO:0003824).

To further reveal the biological functions and the interactions of the gene products, a pathway-based analysis was performed by using the Kyoto Encyclopedia of Genes and Genomes (KEGG) pathway database. KEGG records the molecular interaction networks in cells with variants that are specific to particular organisms. The results showed that 15,239 unigenes were mapped to 231 KEGG pathways for the transcriptomes of HS6, HS9, HS12 and HS20 (Additional file 2: Table S2). The top 3 pathways were PI3K-Akt signaling pathway (ko04151, 561 genes), Calcium signaling pathway (ko04020, 506 genes) and cAMP signaling pathway (ko04024, 496 genes) (Additional file 2: Table S2, yellow mark). The primary pathways involved in gonadal development include GnRH signaling pathway (203 genes), MAPK signaling pathway (ko04010, 476 genes), Wnt signaling pathway (ko04310, 226 genes), Estrogen signaling pathway (ko04915, 220 genes), Progesterone-mediated oocyte maturation (ko04914, 154 genes) and ovarian steroidogenesis (ko04913, 70 genes) (Additional file 2: Table S2, blue mark). Besides, we found that a lot of unigenes were mapped to the main pathways involved in germ cell meiosis, such as oocyte meiosis (ko04114, 199 genes), cell cycle (ko04110, 159 genes), base excision repair (ko03410, 31 genes), DNA replication (ko03030, 30 genes) and homologous recombination (ko03440, 27 genes) (Additional file 2: Table S2, green mark). These pathways may provide good starting points for exploring the ovarian differentiation and development of ricefiled eel larvae in our future research.

### Expression of functional genes putatively related to gonadal differentiation and retinol metabolism

**Fig. 4:**
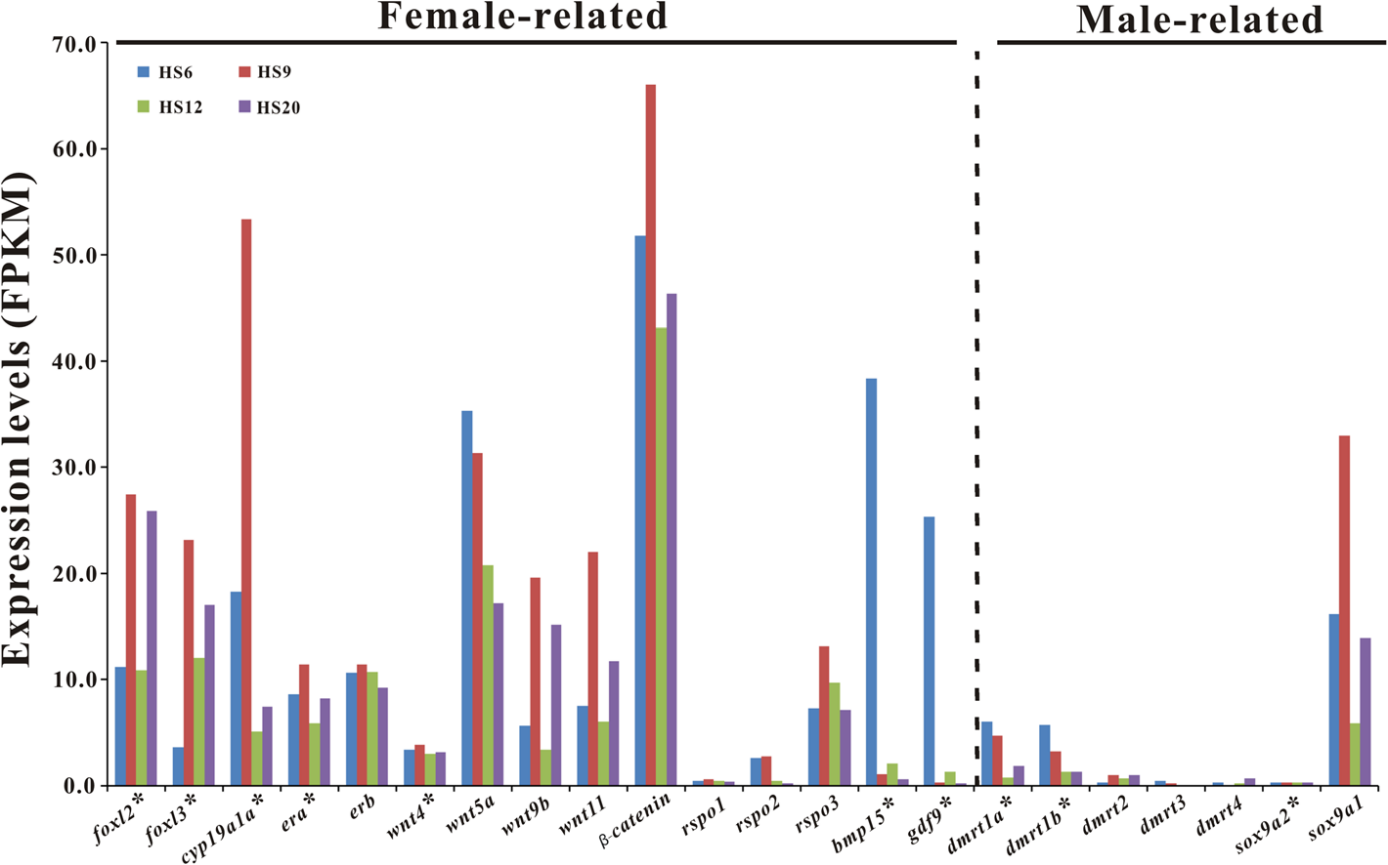
Expression of early gonadal development-related functional genes in gonads of ricefield eel larvae at 6, 9, 12, and 20 dph. Y-axis shows FPKM values of genes inferred from the transcriptome data. The asterisk-marked genes were further analyzed and confirmed by qPCR. HS6: 6 dph; HS9: 9 dph; HS12: 12 dph; HS20: 20 dph

The expression of functional genes putatively related to gonadal differentiation (15 female-related and 7 male-related; Fig. 4) at 6, 9, 12, and 20 dph was analyzed using transcriptome data*.* Twelve of the female-related genes, including *foxl2*, *foxl3*, *cyp19a1a*, *era*, *erb*, *wnt5a*, *wnt9b*, *wnt11*, *β-catenin*, *rspo3*, *bmp15* and, *gdf9* are of relatively high expression at some time points from 6 to 20 dph, with highest FPKM ranging from 11.4 to 66.0. In contrast, all the male-related genes, except *sox9a1*, were of low expression or no expression. Interestingly, all of the female-related genes, expect *wnt5a*, *gdf9* and *bmp15*, had peaked expression at 9 dph. The expression of *wnt5a* was maintained at relatively high levels from 6 to 20 dph while the expression of *gdf9* and *bmp15* was highest at 6 dph, but fell precipitously at 9 dph and remained at low levels thereafter. The other three female-related genes, *wnt4*, *rspo1*, and *rspo2*, maintained relatively low expression levels from 6 to 20 dph, implying that these three genes may not be the major player in the ovarian development of ricefield eel larvae.

**Fig. 5:**
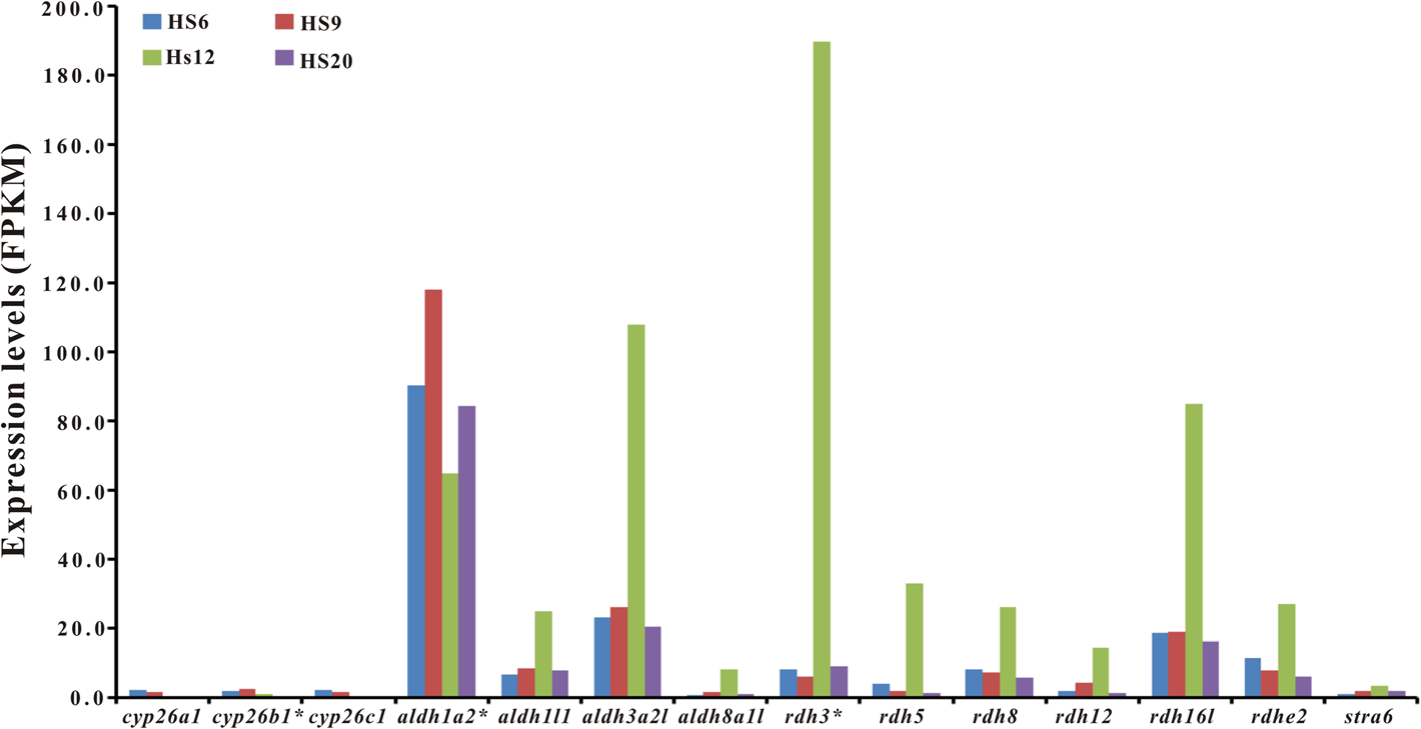
Expression of functional genes of the retinol metabolism pathway in gonads of ricefield eel larvae at 6, 9, 12, and 20 dph. Y-axis shows FPKM values of genes inferred from the transcriptome data. The asterisk-marked genes were further analyzed and confirmed by qPCR. HS6: 6 dph; HS9: 9 dph; HS12: 12 dph; HS20: 20 dph

As retinoic acid (RA) activates germ cells to initiate meiosis and play important roles in gonadal differentiation and development, the expression of genes in the retinol metabolism pathway was analyzed (Fig. 5). For the synthesis of RA, retinol dehydrogenase (Rdh) converts vitamin A into retinal, followed by the conversion of retinal to RA by the catalysis of aldehyde dehydrogenase (Aldh). The degradation of RA is catalyzed by enzymes of the cytochrome P450 family 26 (Cyp26). From the transcriptome data, six *rdh* and four *aldh* genes were identified, together with three *cyp26* genes. All the three *cyp26* genes, *cyp26a1*, *cyp26b1*, and *cyp26c1*, exhibited low expression in the early gonads from 6 to 20 dph, particularly at 12 and 20 dph. The expression of *aldh1a2*, an aldehyde dehydrogenase gene, remained constantly at high levels from 6 to 20 dph. In contrast, the expression of three *aldh* (*aldh1l1*, *aldh3a2l*, *aldh8a1l*) and six *rdh* (*rdh3*, *rdh5*, *rdh8*, *rdh12*, *rdh16l*, *rdhe2*) genes varied greatly with similar patterns, increased substantially at 12 dph but then decreased precipitously at 20 dph. The most dramatic changes were observed in *rdh3* expression, which was increased by 31 folds at 12 dph but decreased by 21 folds at 20 dph. A homologue of *stra8,* a specific pre-meiotic marker could not be identified in the present transcriptome data, however, a homologue of *stra6* was identified and showed a similar expression pattern as those of *rdh* genes, with highest expression at 12 dph.

### Differentially expressed genes (DEGs) in gonads of ricefield eel larvae between 9 dph vs 6 dph, 12 dph vs 9 dph, and 20 dph vs 12 dph

The numbers of DEGs between HS9 (9 dph) and HS6 (6 dph), HS12 (12 dph) and HS9 (9dph), and HS20 (20 dph) and HS12 (12 dph) were 930, 1255 and 1326, respectively (Additional file 1: Fig. S4A-D). There were only 124 common differentially expressed genes among the above three comparisons (Additional file 1: Fig. S4D), implying that most of DEGs were unique to each comparison. Totally, only 175 differentially expressed genes (indicated with a red box) between 9 dph and 6 dph were also differentially expressed between 20 dph and 12 dph (Additional file 1: Fig. S4D), which represents only 27.8% of DEGs in the former comparison and 13.2% of DEGs in the latter and suggests that dramatic developmental changes occurred between stages from 6 to 9 dph and from 12 to 20 dph. Up-regulated genes at 9 dph compared to 6 dph, 12 dph compared to 9 dph, and 20 dph compared to 12 dph were 257, 745, and 389, respectively (Additional file 1: Fig. S4A-C). But down-regulated genes at 9 dph compared to 6 dph, 12 dph compared to 9 dph, and 20 dph compared to 12 dph were 673, 510, and 937, respectively (Additional file 1: Fig. S4A-C).

**Fig. 6:**
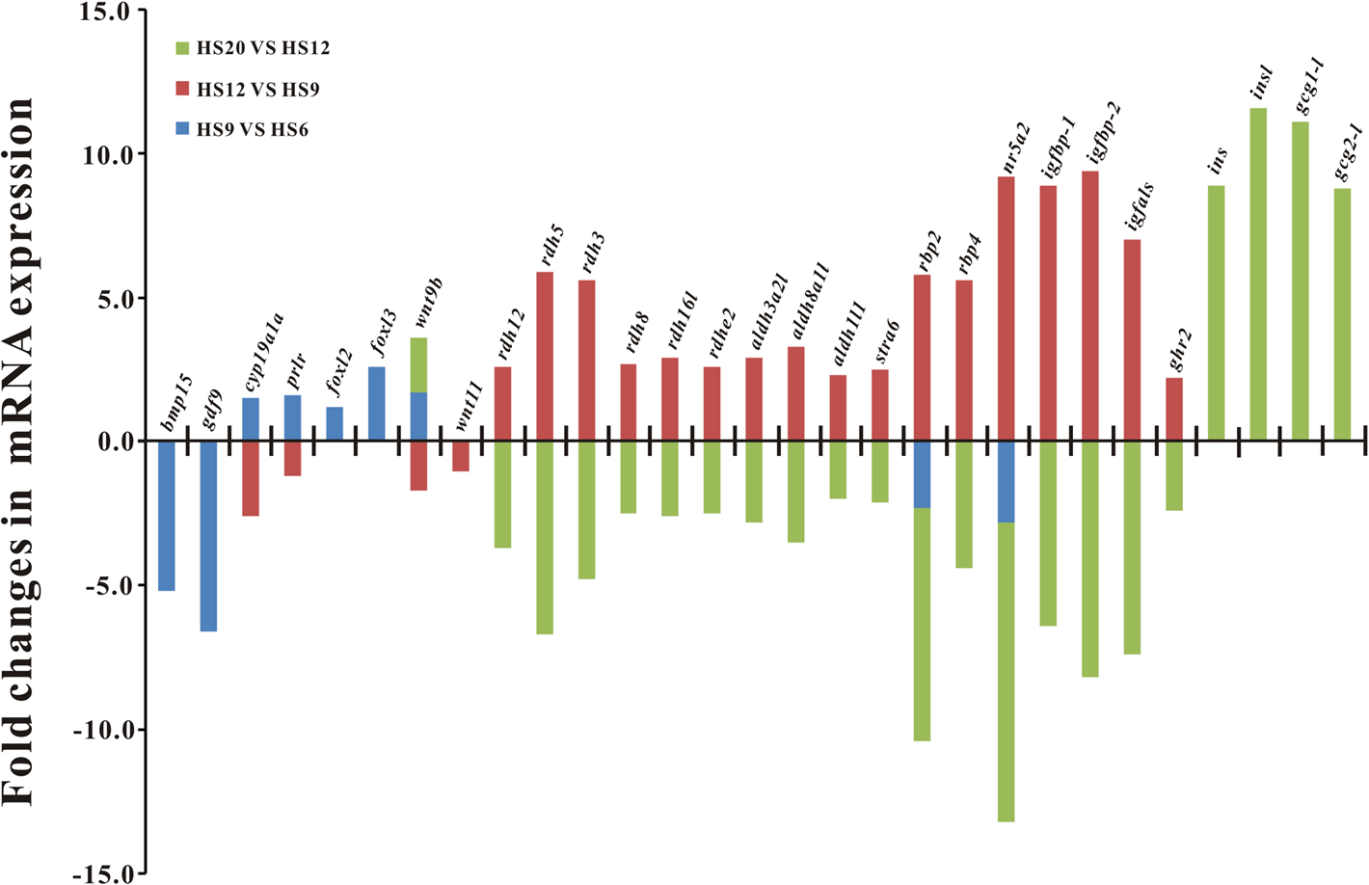
Differential expression of some representative genes during gonadal development of ricefield eel larvae. Y-axis shows the fold changes in mRNA expression of genes in the designated comparison. HS6: 6 dph; HS9: 9 dph; HS12: 12 dph; HS20: 20 dph. The columns above and below X-axis indicate up-regulation and down-regulation, respectively

Of the above selected functional genes related to gonadal differentiation, some of them were found to be differentially expressed during early gonadal differentiation, including *bmp15*, *gdf9*, *cyp19a1a*, *foxl2*, *foxl3*, *wnt9b*, and *wnt11* (Fig. 6). *Bmp15* and g*df9* were found to be more expressed at 6 dph as compared to those at 9 dph (Fig. 6; Additional file 2: Table S3), but they were not DEGs in the comparison between 12 dph and 9 dph or between 20 dph and 12 dph (Additional file 2: Table S5-S8). *Cyp19a1a* and *prlr* (prolactin receptor) were upregulated at 9 dph when compared to that at 6 dph (Fig. 6; Additional file 2: Table S4), and downregulated at 12 dph when compared to that at 9 dph (Fig. 6; Additional file 2: Table S5), but were not DEG in the comparison between 20 dph and 12 dph (Additional file 2: Table S7-S8). Both *foxl2* and *foxl3* were upregulated at 9 dph as compared to 6 dph (Fig. 6; Additional file 2: Table S4), but they were not DEGs in the comparison between 12 dph and 9 dph or between 20 dph and 12 dph (Additional file 2: Table S5-S8). *Wnt9b* was upregulated at 9 dph and 20 dph when compared to those at 6 dph and 12 dph, respectively (Fig. 6; Additional file 2: Table S4 and S8). *Wnt11* was downregulated at 12 dph as compared to 9 dph (Fig. 6; Additional file 2: Table S5), but not a DEG in the comparison between 9 dph and 6 dph or between 20 dph and 12 dph (Additional file 2: Table S3, S4, S7, and S8). The above DEGs may play more important roles in the ovarian differentiation of ricefield eels at the early stages up to 9 dph.

In addition, all the six *rdh*, three *aldh* (*aldh3a2l*, *aldh8a1l*, *aldh1l1*), and *stra6* genes were more expressed at 12 dph when compared to those at 9 dph and 20 dph (Fig. 6; Additional file 2: Table S6 and S7), but not DEGs in the comparison between 9 dph and 6 dph (Additional file 2: Table S3 and S4). Other DEGs that were more expressed at 12 dph when compared to those at 9 dph and 20 dph include *rbp4* (retinol-binding protein 4), *rbp2* (retinol-binding protein 2), *igfbp-1* (insulin-like growth factor binding protein-1), *igfbp-2* (insulin-like growth factor binding protein-2), *igfals* (Insulin-like growth factor-binding protein complex acid labile subunit), *ghr-2* (growth hormone receptor 2), *nr5a2* (Nuclear receptor subfamily 5 group A member 2) (Fig. 6; Additional file 2: Table S6 and S7)*.* The similar expression patterns of these DEGs with those of *rdhs* indicated that they might play roles during meiosis in the ovary of ricefield eel larvae.

Some DEGs were shown to be only upregulated at 20 dph, which include *ins* (insulin), *ins-like* (insulin-like), and *gcg1-like* (glucagon-1-like), and *gcg2-like* (glucagon-2-like) (Fig. 6; Additional file 2: Table S8). These DEGs may also be involved in the ovarian development of ricefield eel larvae.

### qPCR validation of gene expression inferred from transcriptome data

**Fig. 7:**
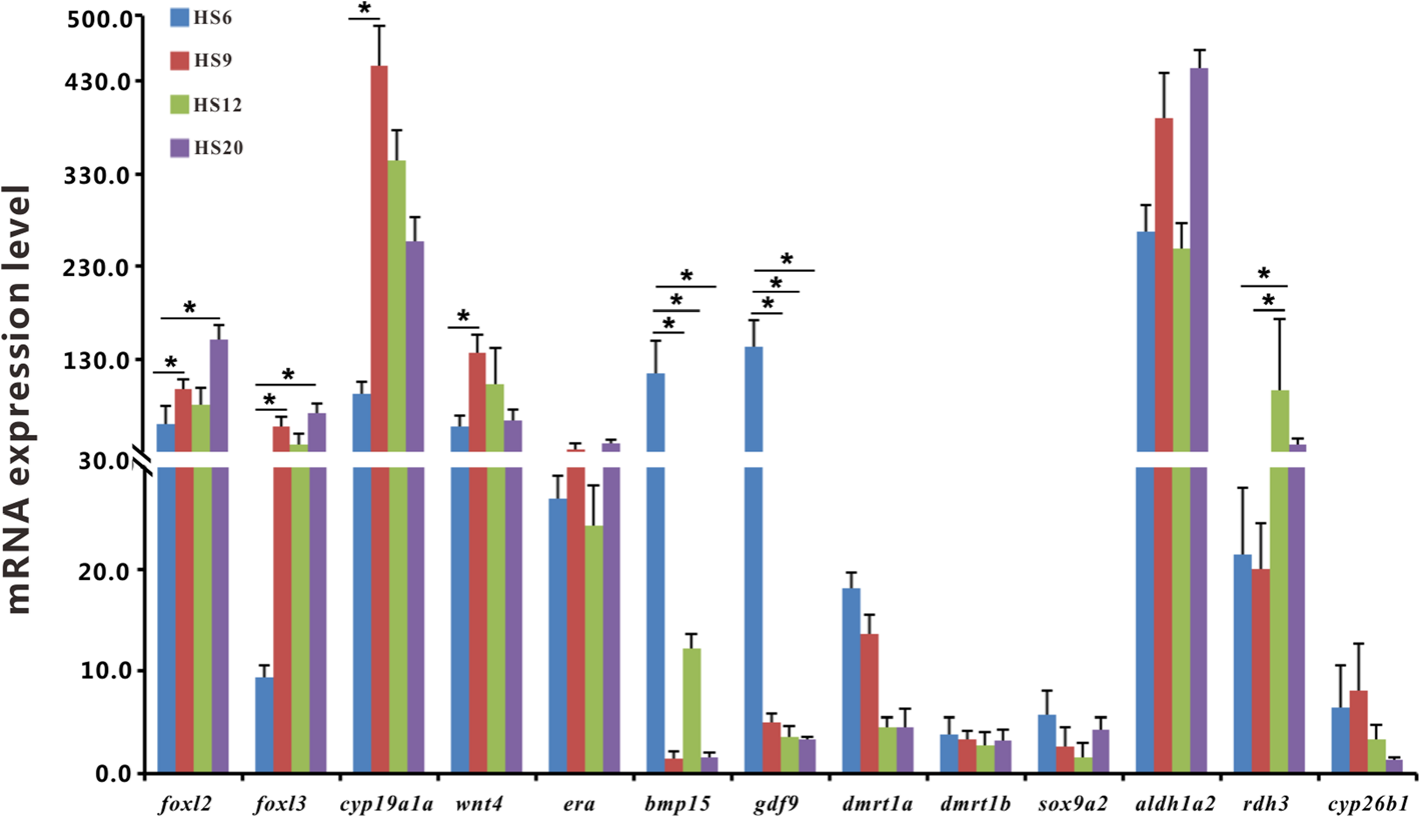
Quantitative PCR validation of the expression of the 13 representative transcripts identified from transcriptome data. The mRNA expression level of the gonad development-related genes was analyzed with qPCR. The data are the average ± standard error (*n* = 3–5). One-way analysis of variance analysis (*P* < 0.05) were used to estimate the significance of gene expression levels. Significant differential expressions of genes between any two stages were marked by a star

qPCR was performed to validate the expression patterns of 13 transcripts (asterisk-marked genes in Figs. 4 and 5) inferred from the transcriptome data, which are presumably associated with sex determination and differentiation. The developmental changes of all the 13 transcripts from 6 to 20 dph as revealed by qPCR analysis (Fig. 7) are basically similar to those inferred from transcriptome data (Additional file 1: Fig. S5), implying that the analysis of gene expression through RNA-seq data in the present study are reliable. The expression of some female-related genes (*foxl2*, *foxl3*, *cyp19a1a*, *wnt4*, *era*, *gdf9*, *bmp15*) was at high levels whereas those of the male-related genes (*dmrt1a*, *dmrt1b*, *sox9a2*) was at low levels. Besides, the mRNA expression of *aldh1a2* and *rdh3* was at high levels whereas that of *cyp26b1* was at low levels from 6 to 20 dph, with peak expression of *rdh3* occurring at 12 dph (Fig. 7).

## Discussion

To the best of our knowledge, the present study is the first comprehensive transcriptomic analysis of ovarian development from undifferentiated to differentiated stages in ricefield eels. This work aimed at identifying genes and regulatory pathways related to the ovarian differentiation and the initiation of meiosis, and eventually explaining how the early undifferentiated gonad of ricefield eel larvae develops into an ovary but not a testis. The dissected gonadal tissues from ricefield eel larvae were confirmed to be authentic by successful amplification of *vasa* gene, and the gonadal developmental stages were verified by histological analysis in the present study. The data obtained may help to improve our understanding on mechanisms underlying sexual differentiation in the ricefield eel as well as other vertebrates, and aid in the development of large-scale artificial breeding techniques for ricefield eel aquaculture.

### Transcriptomic overview of early gonadal development in ricefield eels

To date, genome and transcriptome resources for ricefield eels are extremely limited. The large-scale RNA sequencing performed has generated a wealth of data which is helpful for further understanding the basic biological mechanisms of gonadal differentiation and development, and reproduction in ricefield eels.

In the present study, *de-novo* assembly of the sequencing data from four gonadal cDNA libraries of ricefield eel larvae resulted in 265,896 unigenes, with an average length of 720 bp and a N50 of 1107 bp which were comparable to those documented in some other organisms, such as *O. niloticus* (mean length = 618 bp, N50 = 852 bp) [33], mandarin fish (mean length = 506 bp, N50 = 611 bp) [34]. These results suggest that the quality of sequencing data in the present study is acceptable. Only 71,158 (26.76%) unigenes had at least one significant match with an existing gene model in BLAST searches, which was lower than those reported in mandarin fish (41.6%) [35] and *Synechogobius hasta* (62.71%) [36], and the species distribution for the best match from each sequence showed the highest homology to *Larimichthys crocea* (only 26.3%), followed by *Stegastes partitus* (20.3%), and *Oreochromis niloticus* (7.6%). In the GO analysis, 40,672 (15.29%) unigenes were annotated, which was much less than those reported in *Morone saxatilis* (36.7%) [37]*, Coilia ectenes* (31.27%) [38]. The lower percentage values for the gonadal sequencing data of ricefield eel larvae stated above are probably due to limited number of gene sequences for Synbranchiformes in public databases, and suggest that a great number of new sequences were obtained in the present study.

KEGG pathway analysis showed that 15,239 unigenes were mapped to 231 KEGG pathways for gonadal transcriptomes of ricefield eel larvae. Some biological pathways putatively involved in gonadal development were obtained, including Gnrh signaling pathway, MAPK signaling pathway, Wnt signaling pathway, estrogen signaling pathway, progesterone-mediated oocyte maturation and ovarian steroidogenesis, which is similar to the case in *Scylla paramamosain* [39]. In addition, the present study showed that 199 unigenes were mapped to oocyte meiosis, 159 unigenes mapped to cell cycle, 31 unigenes mapped to base excision repair, 30 unigenes mapped to DNA replication and 27 unigenes mapped to homologous recombination. These pathways may provide good starting points for exploring the ovarian differentiation and development of ricefiled eel larvae in our future research. Moreover, 81.2% DEGs identified between 9 dph and 6 dph were different from those between 20 dph and 12 dph, implying that major molecular events for the ovarian development are different between these two periods of ricefield eel larvae.

### Genes associated with the early ovarian differentiation in ricefield eel larvae

Ricefield eel is a protogynous hermaphrodite teleost and the indifferent gonadal primordium differentiates as an ovary first in juvenile fish and offers a good model for analyzing the mechanisms underlying the ovarian differentiation. Previously, we have examined the ovarian differentiation histologically and results showed that the gonadal primordium of ricefield eels started to differentiate into an ovary at 7 dph [25]. Our present study confirmed that the gonads were indifferent at 6 dph but differentiated into ovaries at 9 dph with the appearance of ovarian cavities. Transcriptome data showed that the expression of *gdf9* and *bmp15* was particularly high in gonadal tissues at 6 dph, but decreased precipitously at 9 dph and remained at low levels thereafter. Gdf9 and Bmp15, members of the transforming growth factor beta (TGFb) superfamily, have been demonstrated to be important in oocyte maturation and folliculogenesis [40, 41]. Bmp2, Bmp4 and Bmp8 are suggested to maintain primordial germ cell (PGC) development in mammals [13]. In addition, 673 (72.4%) DEGs were down-regulated at 9 dph as compared to 6 dph, including cell junction-related proteins, transmembrane proteins, calcium-binding protein, microtubule-associated proteins, low-density lipoprotein and so on (Additional file: 2 Table S2, **yellow mark**). These results suggested that the down-regulated DEGs between 6 and 9 dph including *gdf9* and *bmp15* may be important for the development of the indifferent gonads including PGCs in ricefield eel larvae.

Previously, it has been shown that the expression of *cyp19a1a* and *foxl2* in the gonads of ricefield eel larvae was detectable at earlier stages than those of *dmrt1* isoforms [25]. The transcriptome data of the present study confirmed and extended the above observation by revealing the developmental changes of gene expression at a larger scale around the ovarian differentiation. Foxl2 has been shown to up-regulate *cyp19a* expression in teleosts [42], which presumably enhances the synthesis of estrogen. The transcriptome data showed high expression of *foxl2*, *cyp19a1a*, and *erb* at 9 dph, which coincided with the appearance of the ovarian cavity (a marker for ovarian differentiation) in the present study. These results suggest that estrogen signaling may also play important roles in the ovarian differentiation of ricefield eel larvae, similar to the cases in other teleosts [43].

*Foxl3*, an ancient duplicated copy of *foxl2*, has been shown to be present in teleosts, reptiles, birds, and marsupials [44]. In medaka, adult XX fish with disrupted *foxl3* developed functional sperms in the expanded germinal epithelium of a histologically functional ovary [45], and *foxl3* is considered to be a sex-switching gene that suppresses the initiation of spermatogenesis [46]. In other teleosts like Atlantic salmon and European sea bass, in contrast, *foxl3* transcripts were predominantly expressed in the testis compared to ovaries, and possibly involved in testis physiology [47]. In ricefield eels, a recent study suggests that *foxl3* may be involved in spermatogenesis [47]. Our present study revealed similar developmental changes of *foxl3* as those of *foxl2* and *cyp19a1a* during ovarian differentiation. These results suggest that *foxl3* may also be involved in the ovarian differentiation as well as testicular development in ricefield eels.

In mammals, Wnt/β-catenin pathway has been shown to be crucial for ovarian differentiation, and *β-catenin*, is considered as a female-like gene [48]. In teleosts such as rainbow trout and zebrafish, studies have also implicated Wnt/β-catenin signaling pathway in gonad differentiation [49, 50]. In the gonadal tissues of ricefield eel larvae, 9 forms of *wnt* genes, including *wnt2b*, *wnt4*, *wnt5a*, *wnt5b*, *wnt6*, *wnt7a*, *wnt9a, wnt9b*, and *wnt11*, were identified in transcriptome data. The expression of *wnt5a* was constantly high from 6 to 20 dph and the highest among the nine *wnt* genes. The expression of *wnt9b* and *wnt11* was relatively high and peaked at 9 dph with a similar pattern as that of *cyp19a1a* whereas the expression of other *wnt* genes was relatively low (Additional file 2: Table S9). Interestingly, the expression of *β-catenin* was quite high, being the highest among the female-related genes examined. These results imply that Wnt/β-catenin pathway may play important roles in the ovarian differentiation and development of ricefield eels, and *wnt5a*, *wnt9b*, and *wnt11* presumably had a greater role than other *wnt* genes in this process.

R-spondins function as ligands of the orphan receptors LGR4 and LGR5 to regulate Wnt/β-catenin signaling [51]. In mouse, Rspo1 play crucial roles in female somatic cell differentiation, germ cell commitment to meiosis, and stem cell survival and differentiation by activating the canonical *β-catenin* signaling pathway [52]. In medaka, *Rspo1, 2* and *3* had sexually dimorphic expression profiles with female-specific up-regulation during the critical period of sex determination and differentiation [53]. In our present study, transcriptome analysis showed that *Rspo 3* was expressed at relatively high levels from 6 to 20 dph, with peak expression coinciding with that of *cyp19a1a*, suggesting that *Rspo 3* may play important roles in the ovarian differentiation and development of ricefield eel larvae. In contrast, *Rspo1* and *2* maintained at relatively low levels from 6 to 20 dph, implying that they may not be the major players in the early ovarian differentiation and development of ricefield eel larvae.

*Dmrt1* is an important transcription factor implicated in testicular differentiation [54]. In mice, *Dmrt1* was shown to be necessary for male fertility, Sertoli cell differentiation and maintenance, and germ cell expansion and development [55]. In tilapia, *Dmrt1* deficiency resulted in significant testicular regression; decreased spermatogonia, spermatocytes, and spermatids; physically disabled efferent ducts; and increased abundance of Leydig cells [56]. Other *dmrt* genes have also been implicated in testicular differentiation. In the mouse embryo, *dmrt3* is expressed in the interstitial cells of the developing testis [57]. In medaka and Takifugu, *dmrt3* is expressed in the adult and developing testis but not in the ovary [58, 59]. In ricefield eels, *Dmrt2*, *Dmrt3*, *Dmrt4* and *Dmrt5* are up-regulated during gonad transformation from female to male [60]. In addition, *Sox9* has also been shown to be a critical regulator of the male sex determination pathway in mammals [61]. *Sox9a2,* a duplicated homologue of mammalian *Sox9* in teleosts*,* is initially expressed in somatic cells of both sexes, but upregulated in testicular somatic cells and down regulated in the XX gonads of medaka at 10 to 30 dph [62], suggesting that *sox9a2* may be associated with testicular development in teleosts. In our present study, transcriptome and qPCR analysis revealed very low expression of *dmrt1*, *dmrt2*, *dmrt3* and, *dmrt4*, and sox9a2 in the early gonads of ricefield eel larvae from 6 to 20 dph. Taken together*,* results of the present study suggested that high expression of female-related genes and low expression of male-related genes in early gonads of ricefield eel larvae may promote the indifferent gonads to differentiate into ovaries first. However, further research is required to elucidate the specific roles of these genes in the early gonadal differentiation and development of ricefield eel larvae.

### Genes associated with the initiation of meiosis

Meiosis is essential for the development of germ cells in all sexually reproducing species and is closely related to gonadal differentiation. In mammals, female germ cells enter meiosis earlier than male germ cells, with the former during embryonic development and the latter at puberty [63]. This sex-specific timing of meiosis initiation has been shown to be critically controlled by the presence or absence of the signaling molecule retinoic acid (RA), an active derivative of vitamin A (retinol) [14, 64]. In vertebrates, the level of RA is determined by the balance between its synthesis by Aldh1a retinaldehyde dehydrogenases and its breakdown by Cyp26 Cytochrome P450 enzymes. In chicken and tilapia, studies showed that Aldh1a2 and Cyp26a1 are involved in meiotic initiation of the germ cells [14, 17].

Retinol dehydrogenases (RDHs), members of the short chain dehydrogenase-reductase family, catalyze the conversion of retinol into retinal and play important roles in RA signaling. In zebrafish, knockdown of *rdh1l* results in a robust RA-deficient phenotype including lack of intestinal differentiation, which can be rescued by the addition of exogenous retinoic acid [65]. Our present study showed that the expression of retinol dehydrogenase genes including *rdh3, rdh5, rdh8, rdh12, rdh16l*, and *rdhe2* had peaked at 12 dph, especially prominent for *rdh3* and *rdh5*. In consistency, the expression of aldehyde dehydrogenase genes including *aldh3a2l*, *aldh8a1l*, and *aldh1l1* also peaked at 12 dph and the expression of *aldh1a2* was maintained constantly at high levels from 6 to 20 dph. Interestingly, *rbp2* and *rbp4*, which encodes retinol-binding proteins that bind retinol, also showed peak expression at 12 dph. The presence of presumably abundant retinol-binding proteins in the differentiating ovary at 12 dph may ensure the proper supply of retinol for the synthesis of RA. However, the expression of *cyp26a1, cyp26b1*, and *cyp26c1*, which encode enzymes of the cytochrome P450 family 26 and are responsible for the degradation of RA, were very low from 6 to 20 dph, particularly at 12 dph. In agreement with the notion of Cyp26a1 as the major RA degrading enzyme in teleost [16], the expression of *cyp26a1* but not *cyp26b1* was undetectable at 12 dph. In addition, the expression of *cyp26c1* was also undetectable at 12 dph. The specific expression patterns of the above *rdh*, *aldh*, and *cyp26* genes may be important for the adequate production of RA in the early gonads of ricefield eel, possibly with peak production at 12 dph. Cytological changes of gonadal tissues of ricefield eel larvae indicated that the germ cells had entered meiosis at 20 dph. Thus it could be hypothesized that the possible peak production of RA at 12 dph may trigger the biochemical changes in germ cells and eventually initiate the meiosis around 12 to 20 dph.

Downstream of RA, *stra8* gene is a pre-meiotic marker and is required for meiotic DNA replication and the subsequent processes of meiotic prophase in tetrapods [66]. In teleosts, *stra8* is absent in the genomes of many species, such as stickleback, *Tetraodon*, fugu, medaka, tilapia and zebrafish, although it has been identified recently in catfish [67]. By searching through the gonadal transcriptome data of ricefield eel larvae, *stra6* but not *stra8* was identified. In mammals, Stra6 binds specifically to RBP4 (retinol binding protein 4) and mediates vitamin A uptake by cells [68]. Our present study showed that the developmental changes of *stra6* expression seemed to parallel roughly with those of *rdh* genes. Thus, *stra6* in the early gonads of ricefield eel larvae might promote RA synthesis via a positive feedback mechanism and/or be associated with meiotic initiation, which warrants further studies.

Nr5a2, a member of the nuclear receptor superfamily, has been shown to regulate the expression of genes involved in the metabolism of bile acids [69] and steroids including up-regulation of *cyp19a1a* in teleosts [70]. *Nr5a2* was shown to be expressed in primitive gonad of flounder [71]. Our transcriptome data showed peak expression of *nr5a2* at 12 dph, coinciding with the peak expression of genes involved in RA synthesis in the present study. These results suggest that Nr5a2 is probably involved in the transcription regulation of gene(s) in RA synthesis pathway.

### Genes of endocrine functions in the ovarian differentiation and development of ricefield eel larvae

Prolactin is mainly synthesized and secreted by lactotrope cells of the pituitary and plays important roles in lactation and reproduction by binding to its membrane receptor (Prlr) in different target tissues [72]. *Prlr*−/− female mice are completely infertile with impaired steroidogenesis in the ovary [73]. In teleosts, *prlr* mRNA and/or Prlr proteins have been detected in gonads of several species, and Prl is involved in steroidogenesis and gonadogenesis in both testes and ovaries [74]. In the guppy *Poecilia reticulata*, prolactin was shown to stimulate estradiol-17β synthesis in vitro in vitellogenic oocytes [75]. Our present transcriptome data indicated that the expression of *prlr* peaked at 9 dph, with a change in expression pattern similar to that of *cyp19a1a*. These results suggest that Prl signaling pathway may be involved in the ovarian differentiation of ricefield eel larvae possibly through regulating estradiol synthesis.

In addition to the regulation of growth and metabolism, growth hormone (Gh) is also involved in the modulation of steroidogenesis, gametogenesis, and gonadal differentiation in mammals [76]. In tilapia, the mRNA level of growth hormone receptor (*ghr*) was ten times higher in immature oocytes than those of later stages, suggesting a role for Gh in early stages of oogenesis [77]. Our present study showed that the expression of growth hormone receptor 2 (*ghr2*) peaked at 12 dph after the ovarian differentiation, which is in line with the role of Gh in early stages of oogenesis as suggested [77]. Gh works either directly on target cells or indirectly through its key mediator Igf-I, which is produced primarily in the liver but also in various tissues [78]. Interestingly, the expression of *igfbp1*, *igfbp2*, and *igfals*, components of Igf system [79], also peaked at 12 dph, with a change in pattern similar to that of *ghr2*. Although *gh* transcripts were not identified in the present transcriptome data, the synthesis of Gh in the pituitary of ricefield eel larvae has been shown to start around 3 dph [80]. These results suggest that Gh released from the pituitary of ricefield eel larvae may exert its effects on the early stage of oogenesis possibly through the activation of Igf signals.

Insulin (encoded by *ins*) is mainly produced by beta cells of the pancreatic islets and involved in the regulation of metabolism of carbohydrates, fats and proteins [81]. In addition, in vitro, insulin was also shown to stimulate proliferation of the granulosa cells from bovine follicles in a concentration dependent manner [82], and improve follicular and oocyte growth of preantral caprine follicles in the presence of Gh [83]. Preproglucagon (encoded by *gcg*) is processed to glucagon in pancreatic islet α cells while to other products including GLP-1 and GLP-2 in intestinal L cells [84]. The expression of glucagon receptor and glucagon-like peptide-1 receptor was detected in the ovary of rat [85, 86]. Glucagon receptor mRNA was also present in the ovary of chicken [87]. These results suggest that glucagon or its derivatives may act directly in the ovary of vertebrates. The transcriptome data of our present study identified two forms of preproinsulin genes (*ins*, *insl*) and preproglucagon (*glp1–1*, *glp2–1*) genes. All these transcripts were upregulated at 20 dph, with fold change >8 when compared to those at 12 dph. These results suggest that insulin and glucagon or glucagon derivatives may be produced in the ovary of ricefield eel larvae, and play important roles via paracrine and/or autocrine manners in the ovarian development after initiation of meiosis.

## Conclusions

To our knowledge, our present study is the first report of large-scale RNA sequencing of the early gonads of ricefiled eel larvae. Results of our present study suggest that high expression of female development-related genes and low expression of male development-related genes in the early gonads of ricefield eel larvae participate in the cascade of sex differentiation leading to the final female phenotype. The peak expression of *rdh* and *aldh* genes at 12 dph and continuously low expression of the three *cyp26s* from 6 dph to 20 dph may ensure peak production of RA around 12 dph, which in turn induces biochemical changes inside germ cells for the initiation of meiosis. *Stra6* might be a downstream target of RA in the early gonads of ricefield eel larvae and probably associated with RA metabolism and/or the initiation of meiosis. Data of present study could lay a good foundation for further unraveling the mechanisms underlying the ovarian differentiation in ricefield eels and other teleosts as well.

## Additional files

Additional file 1: **Fig. S1.** The workflow of dissecting out gonads from ricefield eel larvae for RNA extraction. A) Decollating the head with syringe needles. B) Tearing the abdomen open up to the cloacal orifice. C) Pulling out the visceral mass. D) Stripping tissues and organs of the visceral mass. E) Identifying the gonadal tissues. F) Putting the gonadal tissues into the TRK Lysis Buffer (a Lysis Buffer of the E.Z.N.A. MicroElute Total RNA Kit). Scale bar = 1000 μm. **Fig. S2.** RT-PCR analysis of *vasa* (A) and *bactin* (B) expression in the isolated gonadal tissues from ricefield eel larvae*.* The number of PCR cycles was 34. dph, days post hatching; M, DNA marker 3 (Dongsheng, Guangzhou, China); NC, negative control (water was as the template); bp, base pair. **Fig. S3.** Length distribution of unigenes. A) X-axis, the length ranges of unigenes; Y-axis, the number of unigenes in different length ranges. B) X-axis, the unigene length; Y-axis, the number of assembled unigenes of the particular length indicated by the X-axis. The unigene length ranges from 201 bp to 19,212 bp. The average length was 720 bp. The N50 length of unigenes was 1107 bp. The N90 length of unigenes was 289 bp. **Fig. S4.** Volcano plot and Venn diagram of the differentially expressed genes in the gonads. Volcano plot of differentially expressed genes in HS9 vs HS6 (A), HS12 vs HS9 (B), and HS20 vs HS12 (C). Blue splashes refer to genes of expression without significant differences. Red splashes refer to genes significantly up-regulated. Green splashes refer to genes significantly down-regulated. Venn diagram showing the overlaps of the differentially expressed genes (DEGs) in the HS9 vs HS6, HS12 vs HS9, and HS20 vs HS12 (D). HS6: 6 dph; HS9: 9 dph; HS12: 12 dph; HS20: 20 dph. **Fig. S5.** Transcriptome data-derived expression of the 13 transcripts in gonads of ricefield eel larvae at 6, 9, 12, and 20 dph. FPKM: Reads per kilo bases per million mapped reads; HS6: 6 dph; HS9: 9 dph; HS12: 12 dph; HS20: 20 dph (DOC 1812 kb)

Additional file 2: **Table S1.** Sequences of oligonucleotide primers for RT-PCR and qPCR analysis. **Table S2.** List of Unigenes that were mapped to KEGG pathways. **Table S3.** Differentially expressed genes downregulated at 9 dph compared to those at 6 dph. **Table S4.** Differentially expressed genes upregulated at 9 dph compared to those at 6 dph. **Table S5.** Differentially expressed genes downregulated at 12 dph compared to those at 9 dph. **Table S6.** Differentially expressed genes upregulated at 12 dph compared to those at 9 dph. **Table S7.** Differentially expressed genes downregulated at 20 dph compared to those at 12 dph. **Table S8.** Differentially expressed genes upregulated at 20 dph compared to those at 12 dph. **Table S9.** Read counts and FPKM of *wnt* genes (XLSX 1557 kb)

## Abbreviations

Aldh: Aldehyde dehydrogenase
BLAST: The basic local alignment search tool
cDNA: Complementary DNA
CYP26: Cytochrome P26
DEG: Differentially expressed gene
dNTP: Deoxy-ribonucleoside triphosphate
dph: Day post hatching
FPKM: Reads per kilo bases per million mapped reads
Gh: Growth hormone
Ghr2: Growth hormone receptor 2
GO: Gene Ontology
KEGG: Kyoto encyclopedia of genes and genomes database
KO: Kyoto encyclopedia of genes and genomes ortholog database
KOG/COG: EuKaryotic ortholog groups/clusters of orthologous groups of proteins
LGR4: Leucine rich repeat containing G protein-coupled receptor 4
LGR5: Leucine rich repeat containing G protein-coupled receptor 5
N50: N50 statistic defines assembly quality. Given a set of contigs, each with its own length, the N50 length is defined as the shortest sequence length at 50% of the genome
Nr: Non-redundant protein sequences
Nt: NCBI nucleotide sequences
Pfam: Protein family
PGC: Primordial germ cell
Prl: Prolactin
Prlr: Prolactin receptor
Q20: Percentage of bases with a Phred value of at least 20
Q30: Percentage of bases with a Phred value of at least 30
RA: Retinoic acid
Rdh: Retinol dehydrogenase
RT-PCR: Reverse transcriptase-polymerase chain reaction
RT-qPCR: Real-time quantitative PCR
STRA6: Stimulated by retinoic acid 6
SwissProt: A manually annotated and reviewed protein sequence database
TGFβ: Transforming growth factor beta
TGICL: A software system for fast clustering of large EST datasets
