# Supplementary material for: Transcriptomic analysis of the differentiating ovary of the protogynous ricefield eel Monopterus albus
Source: BMC Genomics. 2017 Aug 3;18:573. doi: 10.1186/s12864-017-3953-6 (PMC5541746; doi:10.1186/s12864-017-3953-6)
Supplement: Supplementary file 1 — Fig. S1. The workflow of dissecting out gonads from ricefield eel larvae for RNA extraction. A) Decollating the head with syringe needles. B) Tearing the abdomen open up to the cloacal orifice. C) Pulling out the visceral mass. D) Stripping tissues and organs of the visceral mass. E) Identifying the gonadal tissues. F) Putting the gonadal tissues into the TRK Lysis Buffer (a Lysis Buffer of the E.Z.N.A. MicroElute Total RNA Kit). Scale bar = 1000 μm. Fig. S2. RT-PCR analysis of vasa (A) and bactin (B) expression in the isolated gonadal tissues from ricefield eel larvae. The number of PCR cycles was 34. dph, days post hatching; M, DNA marker 3 (Dongsheng, Guangzhou, China); NC, negative control (water was as the template); bp, base pair. Fig. S3. Length distribution of unigenes. A) X-axis, the length ranges of unigenes; Y-axis, the number of unigenes in different length ranges. B) X-axis, the unigene length; Y-axis, the number of assembled unigenes of the particular length indicated by the X-axis. The unigene length ranges from 201 bp to 19,212 bp. The average length was 720 bp. The N50 length of unigenes was 1107 bp. The N90 length of unigenes was 289 bp. Fig. S4. Volcano plot and Venn diagram of the differentially expressed genes in the gonads. Volcano plot of differentially expressed genes in HS9 vs HS6 (A), HS12 vs HS9 (B), and HS20 vs HS12 (C). Blue splashes refer to genes of expression without significant differences. Red splashes refer to genes significantly up-regulated. Green splashes refer to genes significantly down-regulated. Venn diagram showing the overlaps of the differentially expressed genes (DEGs) in the HS9 vs HS6, HS12 vs HS9, and HS20 vs HS12 (D). HS6: 6 dph; HS9: 9 dph; HS12: 12 dph; HS20: 20 dph. Fig. S5. Transcriptome data-derived expression of the 13 transcripts in gonads of ricefield eel larvae at 6, 9, 12, and 20 dph. FPKM: Reads per kilo bases per million mapped reads; HS6: 6 dph; HS9: 9 dph; HS12: 12 dph; HS20: 20 dph (DOC 1812 kb) [file 12864_2017_3953_MOESM1_ESM.doc]

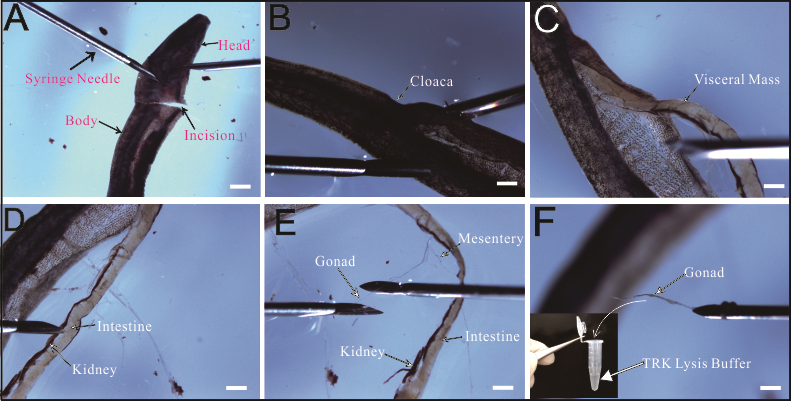


**Figure S1.** The workflow of dissecting out gonads from ricefield eel larvae for RNA extraction. A) Decollating the head with syringe needles. B) Tearing the abdomen open up to the cloacal orifice. C) Pulling out the visceral mass. D) Stripping tissues and organs of the visceral mass. E) Identifying the gonadal tissues. F) Putting the gonadal tissues into the TRK Lysis Buffer (a Lysis Buffer of the E.Z.N.A. MicroElute Total RNA Kit). Scale bar = 1000 μm.


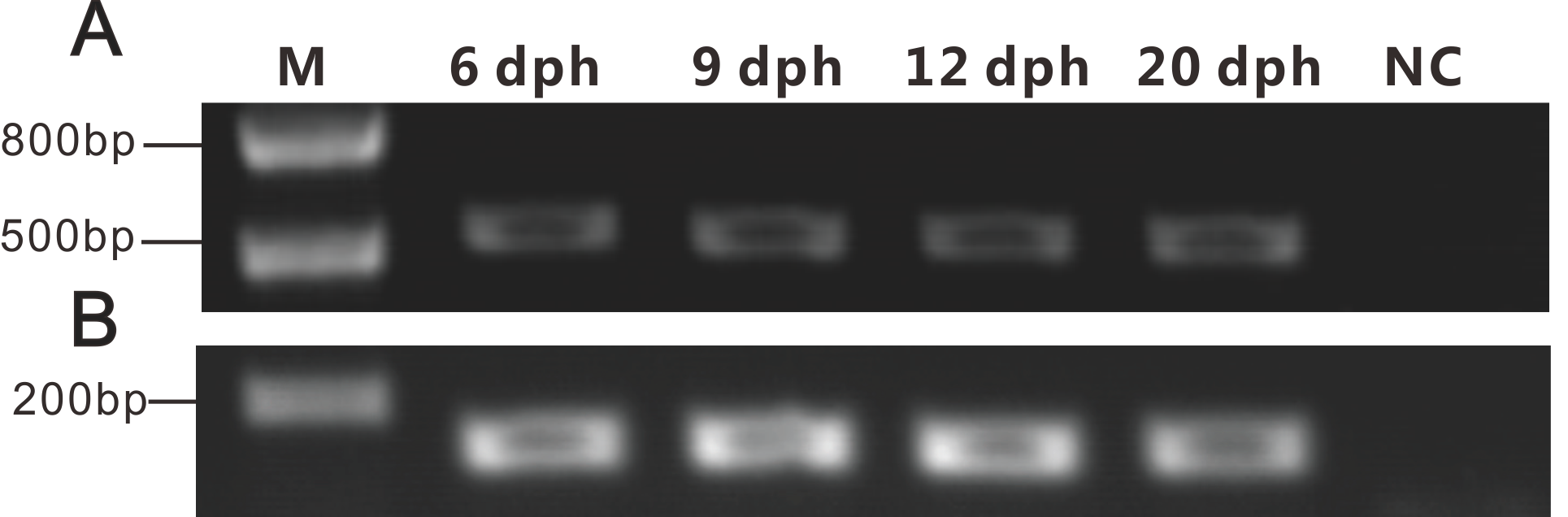


**Figure S2.** RT-PCR analysis of *vasa* (A) and *bactin* (B) expression in the isolated gonadal tissues from ricefield eel larvae*.* The number of PCR cycles was 34. dph, days post hatching; M, DNA marker 3 (Dongsheng, Guangzhou, China); NC, negative control (water was as the template); bp, base pair.


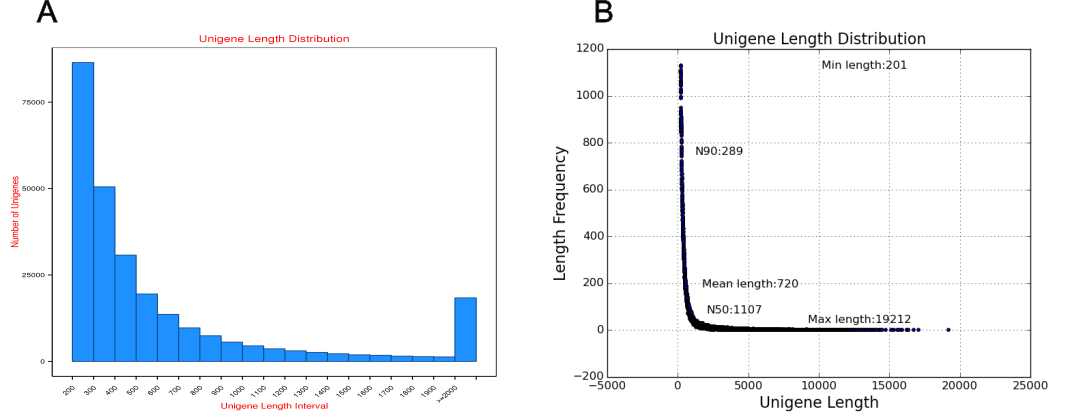


**Figure S3**. Length distribution of unigenes. A) X-axis, the length ranges of unigenes; Y-axis, the number of unigenes in different length ranges. B) X-axis, the unigene length; Y-axis, the number of assembled unigenes of the particular length indicated by the X-axis. The unigene length ranges from 201 bp to 19212 bp. The average length was 720 bp. The N50 length of unigenes was 1107 bp. The N90 length of unigenes was 289 bp.


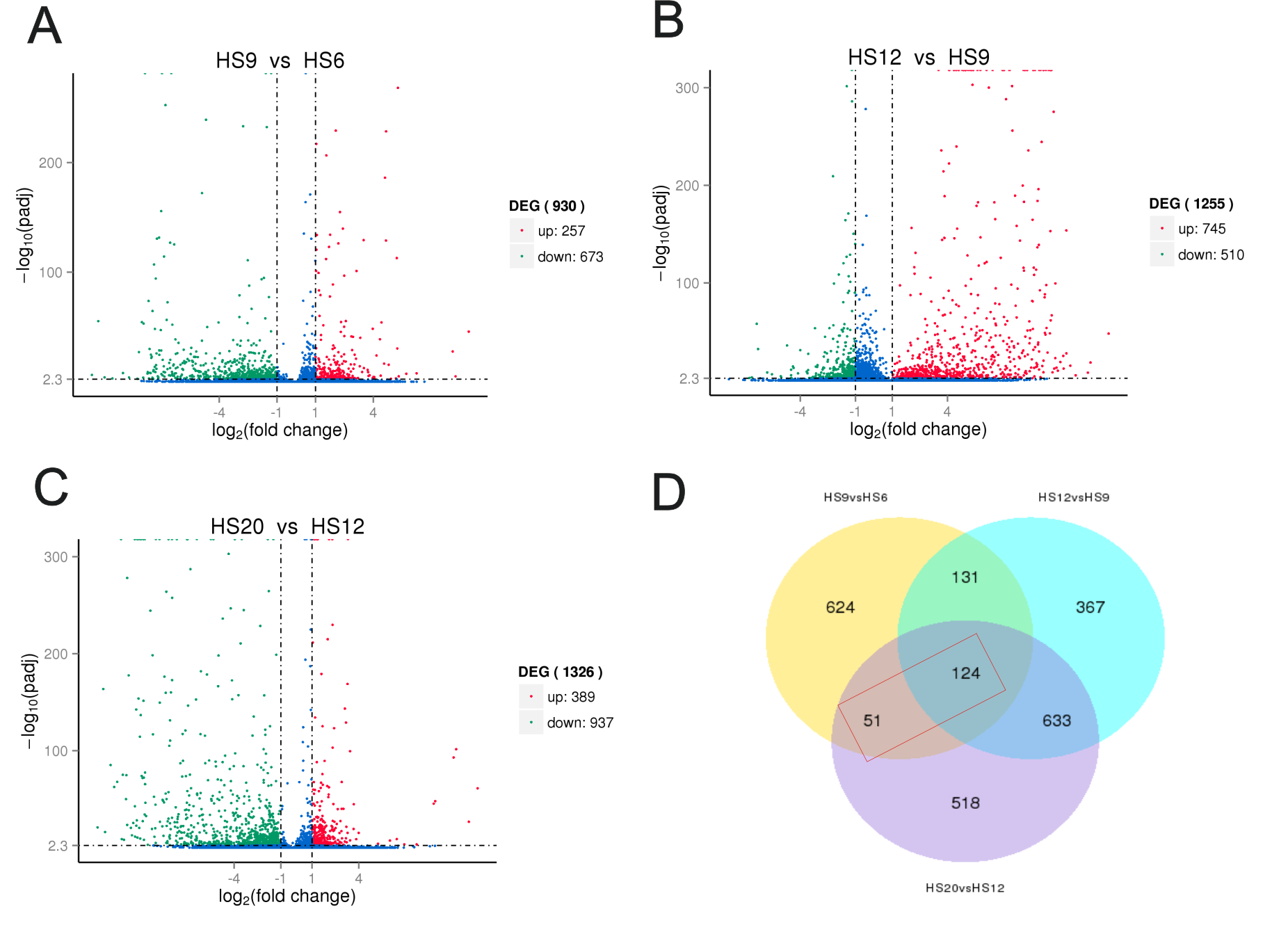


**Figure S4**. Volcano plot and Venn diagram of the differentially expressed genes in the gonads. Volcano plot of differentially expressed genes in HS9 vs HS6 (A), HS12 vs HS9 (B), and HS20 vs HS12 (C). Blue splashes refer to genes of expression without significant differences. Red splashes refer to genes significantly up-regulated. Green splashes refer to genes significantly down-regulated. Venn diagram showing the overlaps of the differentially expressed genes (DEGs) in the HS9 vs HS6, HS12 vs HS9, and HS20 vs HS12 (D). HS6: 6 dph; HS9: 9 dph; HS12: 12 dph; HS20: 20 dph.

**
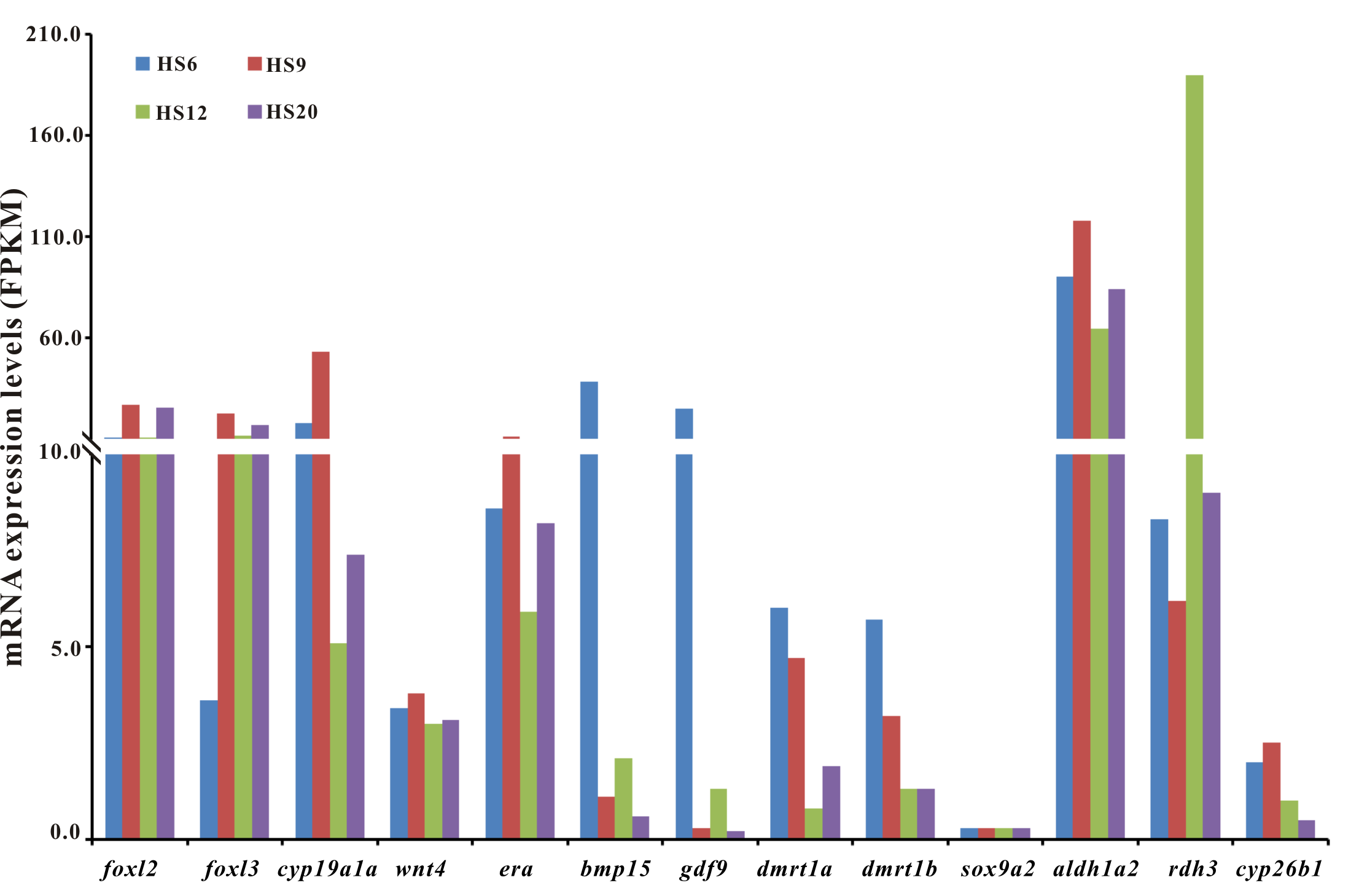
**

**Figure S5**. Transcriptome data-derived expression of the 13 transcripts in gonads of ricefield eel larvae at 6, 9, 12, and 20 dph. FPKM: Reads per kilo bases per million mapped reads; HS6: 6 dph; HS9: 9 dph; HS12: 12 dph; HS20: 20 dph.
